# Overexpression of α-Klotho isoforms promotes distinct Effects on BDNF-Induced Alterations in Dendritic Morphology

**DOI:** 10.1007/s12035-024-04171-y

**Published:** 2024-04-09

**Authors:** Marina Minto Cararo-Lopes, Ratchell Sadovnik, Allen Fu, Shradha Suresh, Srinivasa Gandu, Bonnie L. Firestein

**Affiliations:** 1 Department of Cell Biology and Neuroscience, Rutgers, The State University of New Jersey, Piscataway, NJ USA; 2 Cell and Developmental Biology Graduate Program, Rutgers, The State University of New Jersey, Piscataway, NJ USA; 3 Neuroscience Graduate Program, Rutgers, The State University of New Jersey, Piscataway, NJ USA

**Keywords:** α-Klotho, BDNF, Dendrite branching, TrkB signaling, Calcium, Neuronal development

## Abstract

α-Klotho (α-Kl) is a modulator of aging, neuroprotection, and cognition. Transcription of the *Klotho* gene produces two splice variants—a membrane protein (mKl), which can be cleaved and released into the extracellular milieu, and a truncated secreted form (sKl). Despite mounting evidence supporting a role for α-Kl in brain function, the specific roles of α-Kl isoforms in neuronal development remain elusive. Here, we examined α-Kl protein levels in rat brain and observed region-specific expression in the adult that differs between isoforms. In the developing hippocampus, levels of isoforms decrease after the third postnatal week, marking the end of the critical period for development. We overexpressed α-Kl isoforms in primary cultures of rat cortical neurons and evaluated effects on brain-derived neurotrophic factor (BDNF) signaling. Overexpression of either isoform attenuated BDNF-mediated signaling and reduced intracellular Ca^2+^ levels, with mKl promoting a greater effect. mKl or sKl overexpression in hippocampal neurons resulted in a partially overlapping reduction in secondary dendrite branching. Moreover, mKl overexpression increased primary dendrite number. BDNF treatment of neurons overexpressing sKl resulted in a dendrite branching phenotype similar to control neurons. In neurons overexpressing mKl, BDNF treatment restored branching of secondary and higher order dendrites close, but not distal, to the soma. Taken together, the data presented support the idea that sKl and mKl play distinct roles in neuronal development, and specifically, in dendrite morphogenesis.

**Supplementary Information:**

The online version contains supplementary material available at 10.1007/s12035-024-04171-y.

## Introduction

Expression of the *Klotho* gene is tightly regulated, with highest activity in organs, such as the brain, pituitary gland, kidney, ovary, testis, urinary bladder, skeletal muscle, and pancreas [1, 2]. α-Klotho (α-Kl) protein regulates aging [2, 3], and increased levels of α-Kl in the periphery and CNS are associated with enhancement of cognition and protection against neurodegenerative diseases in animal models and humans [4]. Data regarding the possible roles of α-Kl in development and baseline cell function are scarce in the literature, especially regarding neural cells. Nevertheless, the characterization of α-Kl thus far has implicated this protein in several signaling pathways that play pivotal roles in regulating neuronal development, function, and neuroprotection [4].

**Fig. 1:**
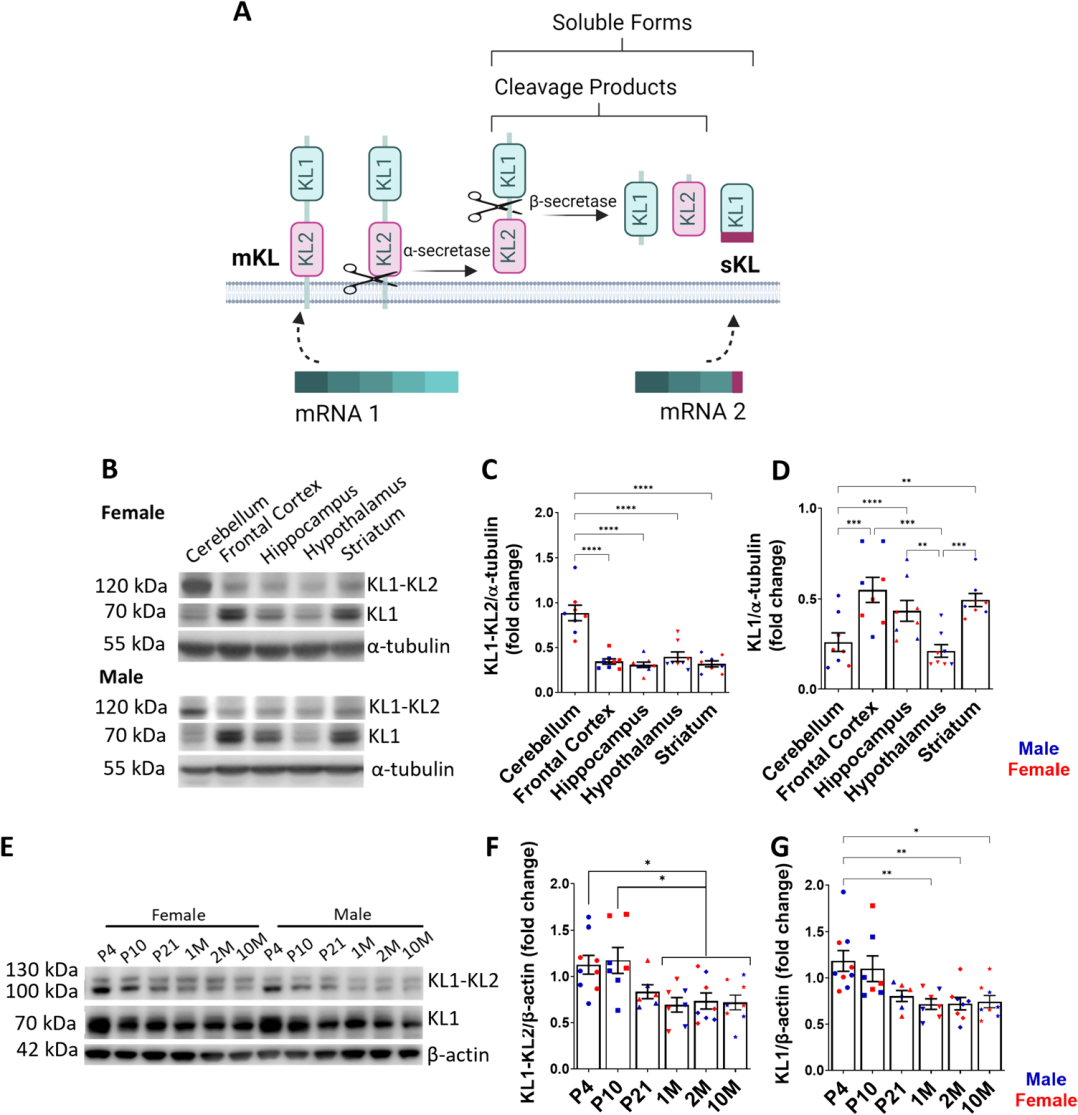
Expression of α-Kl protein in the adult rat brain and in the hippocampus during postnatal development. **A** The *Klotho* gene generates two different transcripts. mRNA 1 is translated into the membrane form of α-Klotho (mKL), which has a transmembrane domain, KL1, and KL2 domains. mKL can be cleaved by α- and β-secretases resulting in the production of cleavage products containing KL1 or KL2 or both domains. The product of alternative splicing (mRNA 2) is translated into secreted α-Klotho (sKl), which contains the KL1 domain and has an additional C-terminal sequence. Created with BioRender.com. **B-D** Full length α-Klotho (KL1-KL2) is expressed at highest levels in the cerebellum while short α-Klotho (KL1) is highest in forebrain regions and striatum. **B** Representative Western blot images of α-Klotho in adult rat brain regions. **C** Quantification of full-length α-Klotho (120 kDa) and (**D**) short α-Klotho (72 kDa) relative to levels of α-tubulin and expressed as fold change of positive control (COS-7 cells transfected with mKl or sKl plasmids). Data are presented as mean ± SEM. **p ≤ 0.01, ***p ≤ 0.001, ****p ≤ 0.0001 as determined by repeated measures one-way ANOVA followed by Tukey’s multiple comparisons test. *n* = 8 animals as represented by individual data points in the graphs. **E–G** α-Klotho levels decline after P21 in hippocampus. **E** Representative Western blot images of full-length mKl (120 kDa) and short isoforms (72 kDa) of α-Klotho in hippocampus across postnatal development. β-actin was used as a loading control. **F** Quantification of full-length α-Klotho (120 kDa) and (**G**) short α-Klotho (72 kDa) levels normalized to β-actin and expressed as fold change of whole brain α-Kl levels. Data are presented as mean ± SEM. *p ≤ 0.05, **p ≤ 0.01 as determined by one-way ANOVA followed by Tukey’s multiple comparisons test. *n* = 6–8 animals per time point as represented by individual data points in the graphs. Abbreviations: P: postnatal day; M: month

α-Kl proteins include different isoforms, which is possibly a factor that contributes to the multiple actions of α-Kl in several signaling pathways. There are two distinct splice variants: a membrane isoform (mKl), containing KL1 and KL2 domains, and a secreted form (sKl), composed of the KL1 domain. mKl protein can be cleaved by α-secretases, such as disintegrin and metalloproteinases ADAM10 and 17, and release a protein containing KL1 and KL2 domains [5–7] (Fig. 1A). The cleavage product can be targeted by β-secretases, producing separate proteins with either KL1 or KL2 domains (Fig. 1A) [5, 7]. One of the pathways implicated in α-Kl-mediated regulation of aging is the inhibition of growth factor-related signaling, particularly IGF-1[3, 8]. The proposed mechanism of action of α-Kl is via modulation of membrane microdomains enriched with sialic acid, such as lipid rafts, that work as signaling hubs for growth factor signaling [9–11]. The KL1 domain has high homology with glucosidases and retains the ability to bind to sialic acid residues present in proteins and lipids, and by doing so, modulates signaling and protein stability [10–12]. The presence of KL2 in mKl physically blocks the sialic acid recognition pocket, impeding this interaction [9–12]. Since the central nervous system (CNS) is enriched in sialic acid-containing gangliosides [13–15], it potentially offers abundant binding sites for α-Kl. Thus, it is plausible that α-Kl expressed in the brain could act via sialic-acid enriched domains to regulate membrane stability and growth factor signaling.

A recent link between α-Kl and another growth factor, brain-derived neurotrophic factor (BDNF) has been reported. For example, serum levels of both α-Kl and BDNF are decreased and correlated in patients with schizophrenia [16]. Similar results have been observed in patients with depressive symptoms [17]. Furthermore, Klotho protein can also be affected by exercise, which elevates BDNF, resulting in higher cognitive function [18] and reviewed in [19]. However, how α-Kl interacts with BDNF signaling and whether the mKl and sKl isoforms have distinct effects on BDNF-mediated signaling is unknown. In the current work, we characterized protein levels of mKl and sKl in rat brain regions and during hippocampal postnatal development. Additionally, we determined the impact of mKl and sKl overexpression on BDNF signaling in cultured cortical neurons. Since BDNF is one of the most-studied regulators of dendrite morphology and synaptic plasticity that has been investigated by our lab and others [20–25], and we observed a peak in the levels of α-Kl isoforms in the hippocampus between postnatal day (P) 4 and 10, when dendritogenesis occurs, we investigated the role of α-Kl and its interaction with BDNF signaling in developing cultured neurons. We found that overexpression of either isoform attenuates BDNF-mediated signaling and reduces intracellular Ca^2+^ levels, and mKl promotes a greater effect. Moreover, overexpression of either isoform reduces higher order dendrites, and only mKl overexpression increases primary dendrite number. sKl-promoted decreases in higher order dendrites are reversed by BDNF treatment, but this change is not observed in neurons overexpressing mKl. Taken together, our data support the idea that sKl and mKl play distinct roles in neuronal development, and specifically, in dendrite morphogenesis.

## Materials and Methods

### Experimental Animals

Adult Sprague Dawley rats of both sexes were obtained from Taconic Biosciences. Timed-pregnant rats were single-housed under a 12 h light–dark cycle and had access to food and water ad libitum. All animal experiments followed ARRIVE guidelines and were approved by Rutgers Institutional Animal Care and Use Committee (protocol # 999900080).

### Adult Rat Brain Dissection

On postnatal days (P) 0, 4, 10, 21, 30, 60, and 10 months of age, rats of each sex were euthanized by CO_2_ inhalation_._ Brain regions of interest (frontal cortex, cortex, hypothalamus, hippocampus, cerebellum, and striatum) were immediately dissected, frozen in dry ice, and stored at -80 °C for later Western blot analysis.

### Primary cortical and hippocampal cultures

Cortices or hippocampi were dissected from rat embryos at 18 days gestation as we previously described (i.e. [23, 26],). Tissue culture plates and glass coverslips were coated with poly-D-lysine (PDL, 0.1 mg/ml) and washed with phosphate-buffered saline (PBS) prior to cell plating. For dendrite branching experiments, hippocampal cells were plated at a density of 5 × 10^4^ cells/cm^2^ on glass coverslips. For Western blot experiments, cortical cells were seeded at a density of 10^5^ cells/cm^2^ in 6 well plates. For cell viability and Ca^2+^ assays, cortical cells used were plated at a density of 10^5^ cells/cm^2^ in a 96 well plate. The cells were cultured in Neurobasal medium (Gibco™, cat# 21103049) supplemented with a final concentration of 10% B-27 (Gibco™, cat# A3582801) and 5% GlutaMAX (Thermo Scientific™, cat# 35050061), which we refer to as full Neurobasal medium. Hippocampal cultures were co-transfected at DIV 7 with a construct expressing mRFP under a CMV promoter and a plasmid containing either GFP (Clontech, pEGFP-C1, control group), mKl-IRES-GFP (VectorBuilder, VB211220-1173gzn), or sKl-IRES-GFP (VectorBuilder, VB211220-1169ryc) using the calcium phosphate method as we previously described [23] using the reagents from CalPhos Mammalian Transfection Kit (Takara Bio USA, Inc., catalog number 631312).

### AAV Transduction of Cortical Neurons

On DIV7, overexpression of sKl or mKl was induced in cortical neurons via transduction with adeno-associated virus (AAV). We used constructs containing the synapsin promoter, which results in neuronal-specific expression of the construct [27]. Plasmid subcloning and ultra-purification of viral particles were performed by VectorBuilder. The sequence for human sKl (NM_153683.2) or mKl (NM_004795.4) followed by a 3X GGGGS linker and a c-Myc tag were subcloned into pAAV vector containing a synapsin promoter (pAAV-Syn-mKl – ID# VB210329-1088sfx; pAAV-Syn-sKl – ID# VB210329-1086xzs). The c-Myc tag allows for identification of successful expression of recombinant α-Kl. This tag is placed at the N-terminus and does not affect α-Kl function [28, 29].

An empty construct containing a stuffer sequence that is not transcribed was used in the control groups (pAAV-Syn-stuffer – ID# VB210323-1179xzw). The sequences were packaged into AAV-PHP.eB capsids, which allows for efficient transduction of neurons [30].

We optimized viral transduction based on the instruction guide provided by VectorBuilder and determined that the multiplicity of infection (MOI) of 2 × 10^4^ viral particles per cell was optimal for our experimental conditions. Briefly, at DIV 7, half of the medium volume was collected and stored, and the cells were transduced with AAV. At 24 h after addition of virus, transduction medium was replaced with a mixture of 50% fresh full Neurobasal medium and 50% conditioned full Neurobasal medium that was collected before the addition of virus.

### Treatment of Cultures with BDNF

Based on previous studies from our group and others [23, 31–33], hippocampal cultures were treated with 25 ng/mL BDNF treatment for 72 h, a length of time that increases dendrite branching. Briefly, BDNF (Peprotech, catalog number 450–02) was dissolved in PBS and diluted to a final concentration of 25 ng/mL in full Neurobasal medium. The medium containing BDNF was added at DIV 11, and at DIV 14, the cells were fixed, and dendritic arbor morphology was assessed.

Similarly, AAV-transduced cortical cultures were treated with 25 ng/mL of BDNF for two different durations: 1) a 5 min treatment at DIV 11, also referred to as short-term treatment, to investigate possible effects of α-Kl on early signaling events triggered by BDNF, and 2) a 72 h treatment from DIV 11 until DIV 14, referred to as long-term treatment, to investigate the impact of overexpression of α-Kl on later signaling events.

### Immunostaining of Cultures

Cell cultures were fixed with 4% paraformaldehyde (PFA) in PBS, washed with PBS, and blocked with 5% goat serum in PBST (PBS containing 0.1% Triton X-100, and 0.02% NaN_3_). Next, the samples were incubated with primary antibodies for MAP2 (1:1000; BD Biosciences cat# 556320, RRID:AB_396359), GFP (1:500; Thermo Fisher Scientific cat# PA1-9533, RRID:AB_1074893)), and RFP (1:500; Rockland cat# 600–401-379, RRID:AB_2209751)). Then, the fixed cultures were rinsed three times with PBS, incubated with respective secondary antibodies conjugated with Alexa-Fluor® 488, Alexa-Fluor® 555, or Alexa-Fluor® 647 (Invitrogen; 1:1000), and rinsed three times with PBS. The neurons, identified by the presence of MAP2 signal, were imaged using an EVOS-FL fluorescence imaging system (ThermoFisher Scientific) with a 20X objective lens.

### Semi-Automated Sholl Analysis

After image acquisition, MAP2-, GFP-, and RFP-positive cells were used for tracing. The presence of GFP indicates the expression of α-Kl constructs, and the RFP signal was used for tracing dendrites. Neurons were traced using NeuronJ (NIH, Bethesda, MD) and NeuronStudio (Mt. Sinai Medical School, NYC, NY), with the experimenter blinded to the condition. Next, the Bonfire program was used to conduct semi-automated Sholl analysis at 6 μm intervals [34, 35], by employing MATLAB (MathWorks, Natick, MA) to convert and export the data to Excel files. We employed an inside-out labeling scheme to determine dendrite order [23, 32].

### Fluo-4 Calcium Assay

The Fluo-4 Direct™ Calcium Assay Kit (Invitrogen™, cat# F10471) was used to assess intracellular Ca^2+^ levels following the manufacturer’s instructions with minor modifications. Primary cortical cells were plated in 96 well plates with black walls. Cells were transduced at DIV 7 and treated with BDNF at DIV 11 for 5 min or 72 h to determine short- and long-term effects of BDNF treatment, respectively. Fluo-4 reagent was diluted in assay buffer with 2.5 mM probenecid, and 100µL was added to each well. To determine the optimal time of probe incubation, the plate was loaded into a Varioskan™ LUX multimode microplate reader (Thermo Fisher Scientific), and fluorescence was measured every 10 min for one hour (excitation at 494 nm and emission at 516 nm). Fluorescent data was acquired from 5 different locations in each well, and based on these measurements, average fluorescence per well was calculated. We determined that after a 10-min incubation, differences due to treatment can be assessed and longer incubation times lead to signal saturation.

### Protein Extraction and Western Blot Analysis

Proteins from rat brain tissue were isolated by the addition of ice-cold RIPA lysis buffer (50 mM Tris (pH 7.4), 1% NP40, 0.25% sodium deoxycholate, 150 mM NaCl, 1 µM EDTA supplemented with protease and phosphatase inhibitors), mechanical dissociation using glass dounce homogenization, and sonication for 20 s [35]. The tissue lysates were centrifuged at 4 °C at 3000 × *g* for 10 min to remove debris. The supernatant was collected, and protein concentration was determined by Pierce™ BCA protein assay.

Proteins were extracted from cortical cultures plated in 6 well plates. At DIV 14, medium was removed, and the cells were rinsed two times with ice-cold PBS. Proteins were isolated by the addition of 120µL ice-cold RIPA lysis buffer, followed by sonication for 20 s [35]. Total cell lysates were centrifuged at 4 °C at 3000 × *g* for 10 min to remove debris. The supernatant was collected, and protein concentration was determined by Pierce™ BCA protein assay.

Protein extracts (20 µg) were loaded onto NuPAGE™ 4 to 12%, Bis–Tris gels (Invitrogen, cat# NP0335BOX) and resolved by electrophoresis (1 h at 130 V) in MOPS running buffer. The proteins were transferred to a PVDF membrane in NuPAGE™ transfer buffer with 10% methanol for 1 h at 20 V. Next, membranes were blocked in 5% BSA in TBS-T (50 mM Tris, 150 mM NaCl, 0.1% Tween 20, pH 7.6) for 1 h and incubated overnight with primary antibodies diluted in block at 1:1000 (anti-α-Kl, Thermo Fisher Scientific cat# PA5-88,303, RRID:AB_2804814; anti-pTrkB, Millipore cat# ABN1381, RRID:AB_2721199; anti-TrkB, Cell Signaling Technology cat# 4603, RRID:AB_2155125; anti-pERK1/2, Cell Signaling Technology cat# 9106 (also 9106L, 9106S), RRID:AB_331768; anti-ERK, Cell Signaling Technology cat# 4695 (also 4695P, 4695S), RRID:AB_390779; anti-pAkt, Cell Signaling Technology cat# 9271 (also 9271S, 9271L, NYUIHC-310), RRID:AB_329825; anti-Akt, Cell Signaling Technology cat# 9272 (also 9272S), RRID:AB_329827; anti-pCREB, Cell Signaling Technology cat# 9198 (also 9198S, 9198L), RRID:AB_2561044; anti-CREB, Cell Signaling Technology cat# 9104, RRID:AB_490881; anti-PSD-95, Antibodies Incorporated cat# 75–028, RRID:AB_2292909; anti-GluN1, Thermo Fisher Scientific cat# 32–0500, RRID:AB_2533060; anti-GluN2B, Cell Signaling Technology Cat# 4207, RRID:AB_1264223; GluR1, Cell Signaling Technology cat# 13,185, RRID:AB_2732897)), 1:5000 (anti-β-actin, Cell Signaling Technology cat# 3700 (also 3700P, 3700S), RRID:AB_2242334) or 1:10,000 (anti-α-tubulin, Abcam cat# ab6161, RRID:AB_305329). Membranes were washed 5 times with TBS-T and incubated with respective HRP-conjugated secondary antibodies (1:2000) in block for 1 h at room temperature. Finally, membranes were developed with Immobilon ECL Ultra (Millipore), the images were acquired using Licor Odyssey software, and band optical density was determined using Image Studio Lite (LI-COR Biosciences).

### Statistical Analysis

Data were obtained from at least two independent experiments, with a minimum of three biological replicates per experiment. N is indicated in each figure. Data were analyzed with one or two-way ANOVA (GraphPad Prism 9) when appropriate. *p* < 0.05 is statistically significant.

## Results

### α-Kl Isoforms Demonstrate Different Expression Patterns in the Adult Rat Brain

The study that first described the *Klotho* gene and α-Kl protein characterized hypomorphic mice and reported the brain as an important tissue for α-Kl production, and specifically, the choroid plexus [2]. Other reports are consistent with this finding and demonstrate the expression of α-Kl in different brain regions of rodents and primates [1, 36–40]. In rats, early data demonstrated the presence of *Klotho* mRNA in the brain by RT-PCR [36, 41] and by in situ hybridization [36] using probes that detect only the KL2 domain. α-Kl protein has also been detected in rat brain and cultured neurons by immunofluorescence, which does not inform us about the expression of α-Kl isoforms [36]. Thus, we performed Western blot analysis to determine expression patterns of the individual α-Kl isoforms.

In adult rat brain, mKl (KL1-KL2) expression levels are highest in the cerebellum (Fig. 1B, C). Interestingly, the short forms of α-Kl, containing only the KL1 domain, are expressed in highest levels in the frontal cortex, striatum, and hippocampus, and to a lesser extent, in the cerebellum and hypothalamus (Fig. 1B, D). No differences in expression were observed between male and female mice as determined by two-way ANOVA (Fig. 1C, *p*=0.7656; Fig. 1D, *p*=0.2607).

### Hippocampal Levels of α-Kl Protein Decline Three Weeks after Birth

The hippocampal formation undergoes dramatic postnatal developmental changes that alter its transcriptional pattern and function [42]. α-Kl is important for hippocampal function in adult mice [43, 44]; however, whether α-Klotho protein levels vary during hippocampal development is not known. Western blot analysis of α-Kl levels in the hippocampus demonstrates that the full-length and short isoforms are high between postnatal days 4 and 10. The doublet observed is a result of the full-length, uncleaved membrane form and the cleaved form containing KL1 and KL2 domains. A previous study that characterized the cleavage of α-Kl showed that in tissue or cell lysate the band appears as a doublet. In medium, which contains only the cleaved Kl1-Kl2 form, there is a single band [6]. The levels of both isoforms significantly decrease after postnatal day 21 (Fig. 1E-G).

### BDNF Treatment does not Affect Endogenous or Recombinant α-Kl Protein Levels

Neurotrophins, including BDNF, are key regulators of neuronal maturation and dendrite branching [23, 45–47]. Since α-Kl affects growth factor signaling, we evaluated the effects of BDNF treatment on TrkB downstream signaling and dendrite morphology in rat primary neurons overexpressing α-Kl isoforms. To study the effects of α-Kl isoforms on BDNF-TrkB signaling, we constructed AAV vectors carrying a neuron-specific promoter to efficiently transduce neurons [27], followed by mKL or sKL with a c-myc tag. Primary neuronal cultures were treated with BDNF for either 5 min or 72 h with the goal of evaluating the effect of α-Kl in early versus late responses to BDNF.

**Fig. 2:**
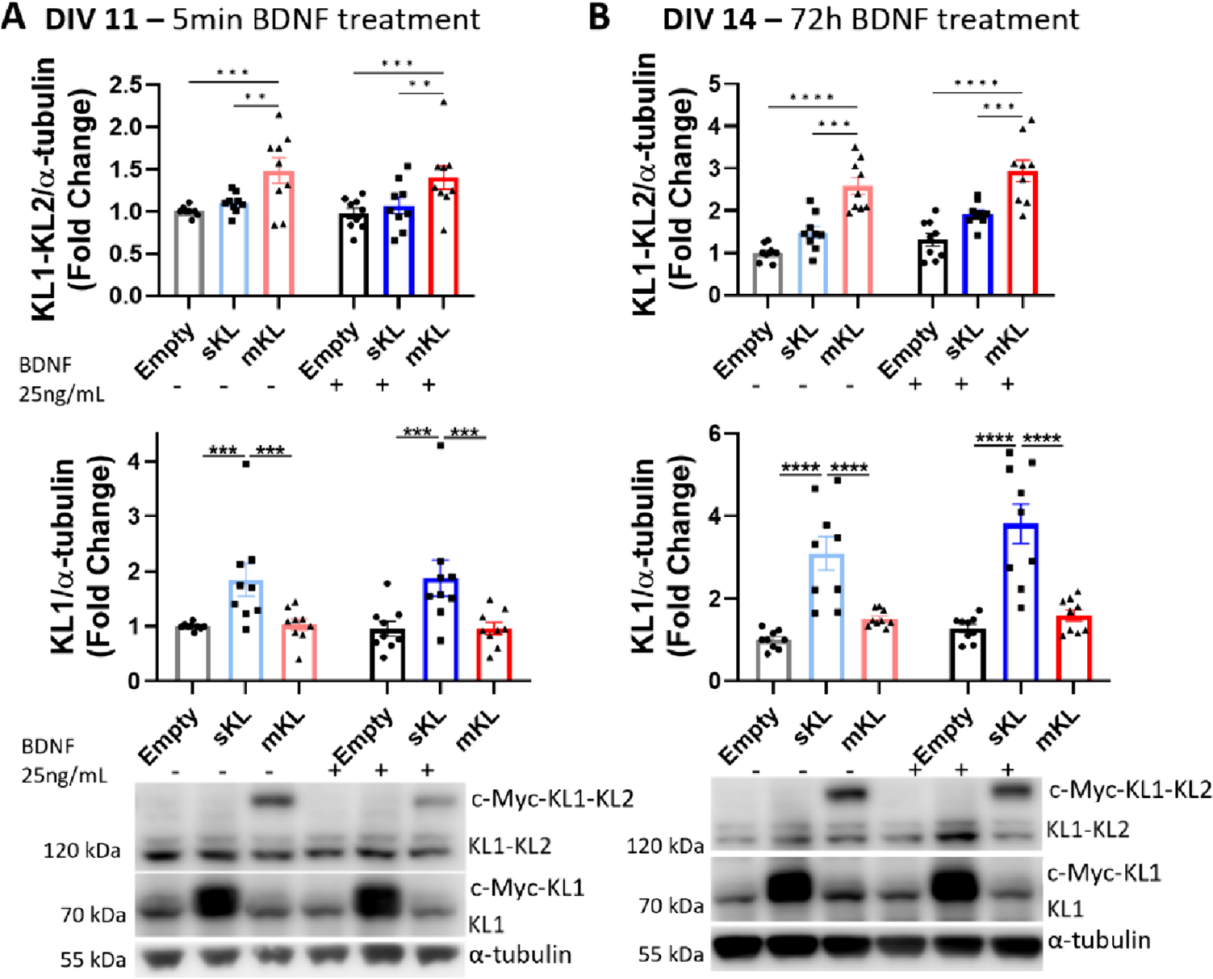
Transduction of neuronal cultures with AAV vectors results in increased α-Kl protein levels, which is unaffected by BDNF treatment. Western blot analysis of α-Kl protein in control cells and cells transduced at DIV 7 with AAV-empty or vector carrying sequences for sKl or mKl expression. Cells were collected on (**A**) DIV 11 after 5 min BDNF treatment and (**B**) DIV 14 after 72 h BDNF treatment. Graphs represent densitometric quantification of mKl (KL1-KL2) and short α-Kl isoforms (KL1) and are followed by respective representative images. Levels of α-Kl were normalized to α-tubulin and expressed as fold change of control average. Data are presented as mean ± S.E.M. *n* = 9 obtained from three independent trials. *p < 0.05, **p < 0.01,***p < 0.001, ****p < 0.0001 as determined by one-way ANOVA followed by Tukey’s multiple comparison test

First, we optimized AAV viral concentration to promote significant sKl and mKl overexpression, which we determined to be 2 × 10^4^ MOI. For each trial, we confirmed the presence of recombinant protein and overexpression of α-Kl by Western blot analysis. Transduction with either sKl or mKl AAV resulted in a significant increase in protein levels of sKl or mKl as detected by expression of KL1 or KL1-KL2 respectively, at DIV 11 (Fig. 2A) and at DIV 14 (Fig. 2B). We observed a greater fold increase of α-Kl expression at DIV 14. Moreover, treatment with 25 ng/mL BDNF for 5 min (Fig. 2A) or 72 h (Fig. 2B) did not significantly impact the expression of endogenous α-Kl isoforms in control neurons transduced with the empty vector, which do not contain the myc-tagged Klotho.

## Overexpression of α-Kl Isoforms Attenuates Increased Intracellular Ca^2+^ Levels Promoted by Long-Term BDNF Treatment*.*

**Fig. 3:**
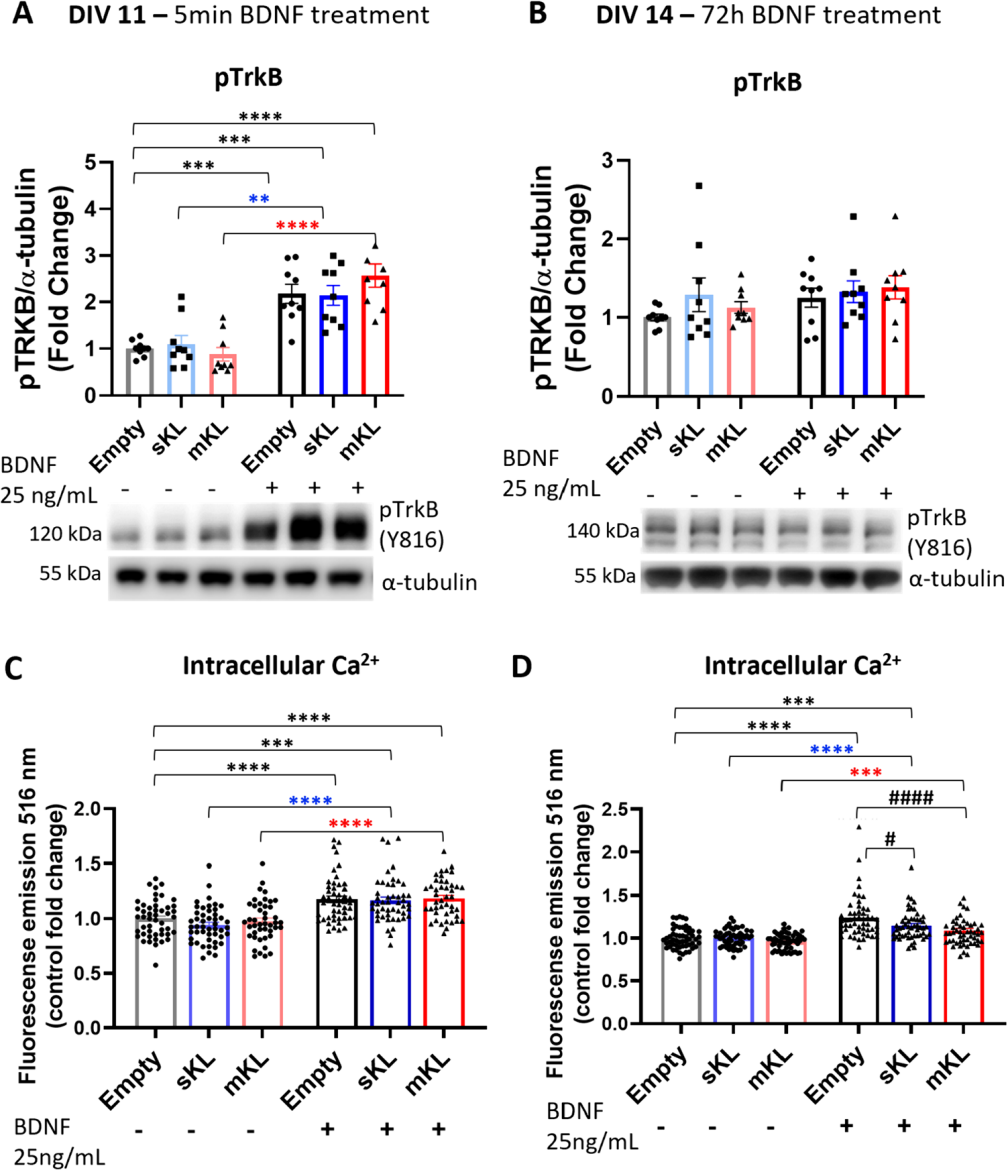
Effects of mKl or sKl overexpression on BDNF-mediated TrkB phosphorylation and increased intracellular calcium levels. BDNF treatment increases TrkB phosphorylation after 5 min treatment, but not 72 h treatment, and this is independent of mKl or sKl overexpression. Cortical cultures were transduced at DIV 7 with AAV-empty, AAV-sKl, or AAV-mKl vectors and treated with BDNF for 5 min or 72 h beginning on DIV 11. **A**, **B** Densitometric quantification and respective representative Western blot images of pTrkB (Y816) levels, which were normalized to α-tubulin levels and expressed as fold change of control (empty, untreated). Data were obtained from three independent trials and are presented as mean ± S.E.M; *n* = 9. **C**, **D** Intracellular Ca^2+^ levels measured by Fluo-4 fluorescence in live cortical cultures. Data were obtained from three independent trials and are presented as mean ± S.E.M; *n* = 52–54. **p < 0.01, ***p < 0.001, ****p < 0.0001, #*p* < 0.05, ####*p* < 0.001 as determined by two-way ANOVA followed by Tukey’s multiple comparisons test: black asterisks, versus untreated AAV-empty control; blue asterisks, versus untreated sKl group; red asterisks, versus untreated mKl group; #, versus BDNF-treated AAV-empty group

As demonstrated previously [48], after 5 min of BDNF treatment, there is a robust increase in TrkB phosphorylation (Fig. 3A), triggering a rise in intracellular Ca^2+^ (Fig. 3C). Additionally, in our experiments, after 72 h BDNF treatment, phosphorylation levels of TrkB return to baseline levels (Fig. 3B). We observed a reduction in the levels of full-length TrkB (140 kDa) in control cultures (AAV-empty group) and in cells overexpressing mKl or sKl with short-term BDNF treatment (Fig. S1). Interestingly, after long-term treatment with BDNF, intracellular levels of Ca^2+^ were higher in the BDNF-treated control (AAV-empty) group when compared to untreated control (Fig. 3D). Cells overexpressing sKl and treated with BDNF demonstrated increased intracellular Ca^2+^ levels when compared to untreated control cultures and untreated sKl overexpressing cells (Fig. 3D). Although BDNF-treated mKl overexpressing cells displayed higher intracellular Ca^2+^ levels when compared to untreated mKl overexpressing cells, no significant differences were observed when comparing versus the untreated AAV-empty control group (Fig. 3D). Finally, both sKl and mKl BDNF-treated groups showed significant reductions in intracellular levels of Ca^2+^ when compared to BDNF-treated control cultures (Fig. 3D). These data suggest that overexpression of either α-Kl isoform decreases Ca^2+^ responses triggered by BDNF and that overexpression of mKl produces a more robust effect.

**Fig. 4:**
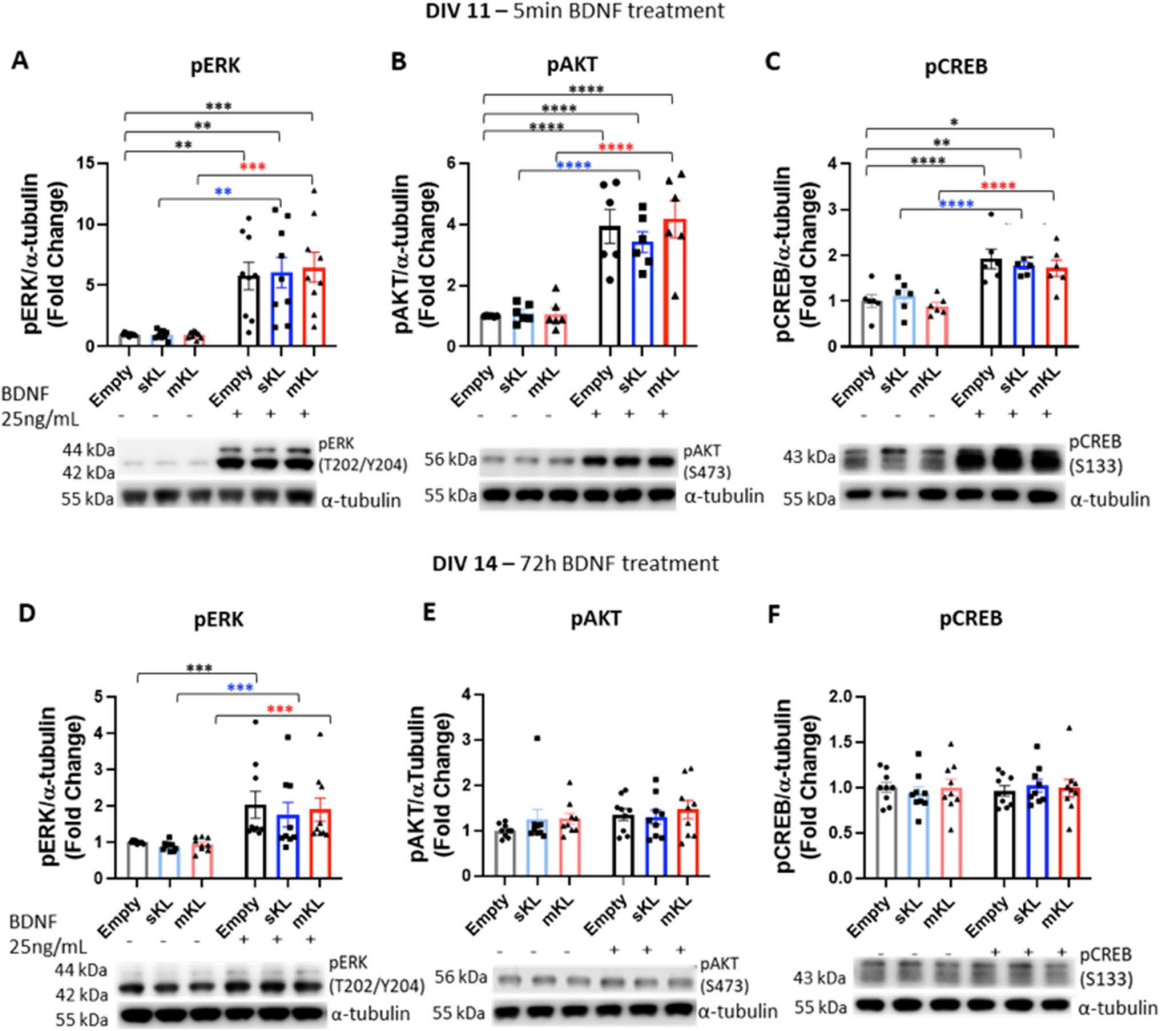
Overexpression α-Kl isoforms does not impact BDNF-mediated ERK, EKT, or CREB phosphorylation. Cortical cultures were transduced at DIV 7 with AAV-empty, AAV-sKl, or AAV-mKl vectors and treated with BDNF for 5 min (**A-C**) or 72 h (**D-F**) on DIV 11. Densitometric quantification and respective representative Western blot images of (**A**, **D**) pERK, (**B**, **E**) pAKT, (**C**, **F**) pCREB levels, which were normalized to α-tubulin levels and expressed as fold change of control (empty, untreated). Data were obtained from three independent trials and are presented as mean ± S.E.M; *n* = 6–9. *p < 0.05, ** *p* < 0.01, *** *p* < 0.001, ****p < 0.0001 as determined by two-way ANOVA followed by Tukey’s multiple comparisons test: black asterisks, versus untreated AAV-empty control group; blue asterisks, versus untreated sKl group; red asterisks, versus untreated mKl group

Activation of TrkB receptors leads to increased phosphorylation of downstream signaling pathways, including ERK, AKT, and CREB, which we observed after 5 min BDNF treatment, and this phosphorylation is independent of α-Klotho overexpression (Fig. 4A-C). After 72 h BDNF treatment, ERK phosphorylation increased in control cultures (Fig. 4D). BDNF also promoted increases in ERK phosphorylation in cells overexpressing mKl or sKl (Fig. 4D). In all groups, levels of AKT and CREB phosphorylation returned to baseline after 72 h BDNF treatment (Fig. 4E,F). Additionally, we did not observe changes to total levels of ERK, AKT, or CREB after BDNF treatment (Fig. S2).

It was previously reported that in mouse cultured hippocampal neurons, AKT phosphorylation is reduced after 24 h incubation with α-Klotho recombinant protein [49]. In primary cultured astrocytes, decreased levels of AKT phosphorylation was also observed, whereas ERK phosphorylation was increased after 15–120 min of incubation with α-Klotho recombinant protein [50]. It is important to note that prolonged exposure to α-Klotho could lead to adaptative changes, which could justify the absence of an effect in these signaling pathways in our experimental model.

### α-Kl Overexpression or 5 min BDNF Treatment does not Affect the Levels of PSD-95, AMPA, and NMDA Receptors

**Fig. 5:**
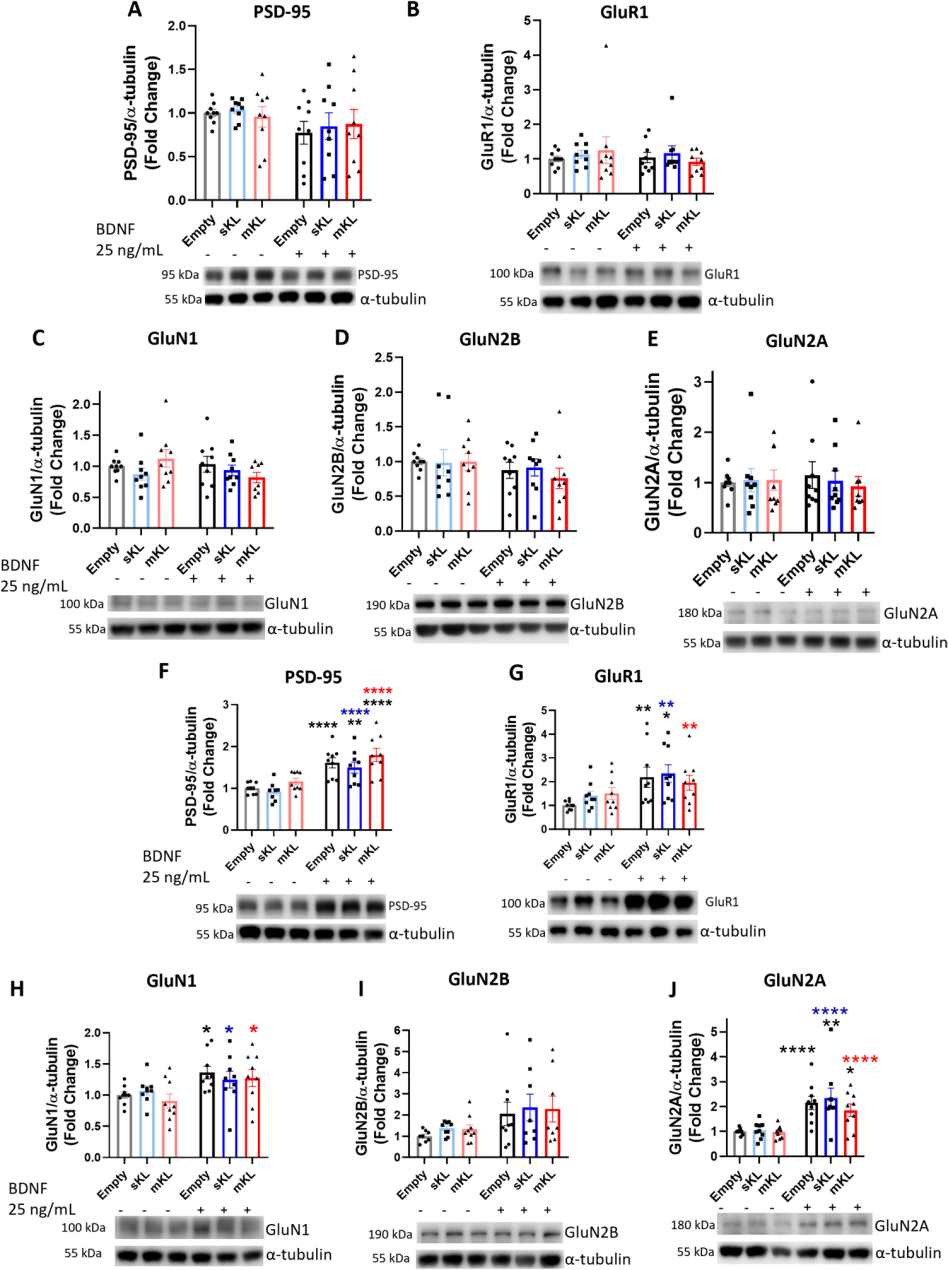
Overexpression of α-Kl isoforms and short-term BDNF treatment does not alter baseline levels of PSD-95, AMPA, and NMDA receptors. Western blot analysis of PSD-95 and AMPA and NMDA glutamate receptor subunits after (A-E) 5 min or (F-J) 72 h BDNF treatment of cortical cells on DIV 11 previously transduced on DIV 7 with AAV-empty, AAV-sKl, or AAV-mKl vectors. Densitometric quantification and respective representative Western blot images of (**A, F**) PSD-95, (**B**, **G**) GluR1, (**C**, **H**) GluN1, (**D**, **I**) GluN2B, and GluN2A (**E**, **J**). Levels of proteins were normalized to α-tubulin and expressed as fold change of control (empty, untreated). Data were obtained from three independent trials and are presented as mean ± S.E.M; *n* = 9. *p < 0.05, **p < 0.01, ****p < 0.0001 as determined by two-way ANOVA followed by Tukey’s multiple comparisons test: black asterisks, versus untreated AAV-empty control group; blue asterisks, versus untreated sKl group; red asterisks, versus untreated mKl group

BDNF treatment increases the levels of synaptic markers and glutamate receptors [51]. Thus, it is important to determine whether overexpression of α-Kl isoforms can alter expression of these proteins prior to and after 5 min treatment with BDNF. We found that neither overexpression of mKl or sKl nor short-term BDNF treatment had an impact on protein levels of PSD-95, AMPA receptors subunit GluR1, or NMDA receptor subunits GluN1, GluN2B, and GluN2A (Fig. 5).

### mKl and sKl Overexpression have Distinct Effects on BDNF-Promoted Changes to the Dendritic Arbor

**Fig. 6:**
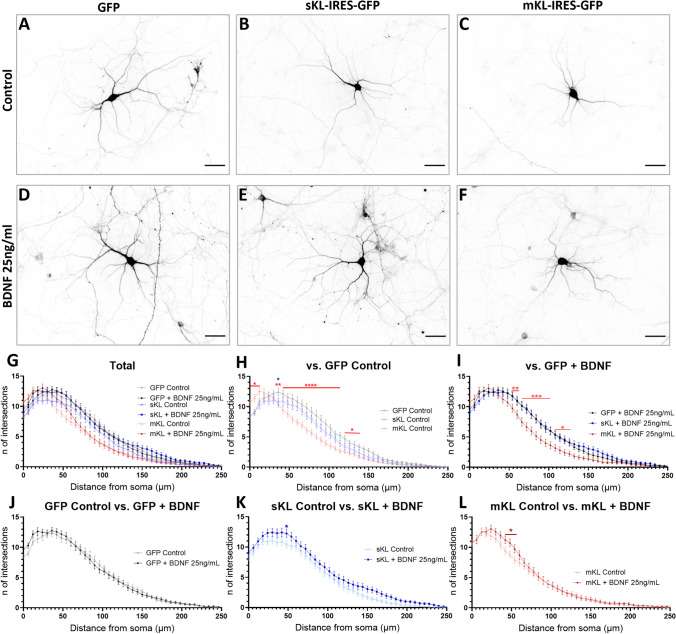
BDNF Treatment Rescues Changes to Dendritic Branching in Hippocampal Neurons Overexpressing sKl but not mKl. Sholl analysis of neurons at DIV 14 that were transfected with empty, sKl, or mKl constructs. Cultures were untreated or treated with 25 ng/mL BDNF for 72 h. **A-F** Representative images of neurons in each experimental condition. Scale bar = 50 µm. **G-L** Sholl curves for dendrites of all orders combined. **G** All groups plotted. **H** Untreated groups. **I** Treatment with BDNF. **J** Control (empty) group without and with BDNF treatment. **K** BDNF effects on cells overexpressing sKl. **L** BDNF effects on neurons overexpressing mKl. Data are presented as mean ± S.E.M; *n* = 41–59. *p < 0.05, **p < 0.01, ***p < 0.001, ****p < 0.0001 as determined by two-way ANOVA followed by Tukey’s multiple comparisons test in which black asterisks indicate comparison versus untreated control (empty) group; blue asterisks versus sKl group; red asterisks versus mKl

Our data suggest that overexpression of sKl and mKl impacts BDNF-triggered Ca^2+^ signaling in neurons. Thus, we asked whether increasing the levels of α-Kl affects changes to the dendritic arbor promoted by 72 h BDNF treatment (25 ng/mL) in rat hippocampal neurons. For the overall dendrite branching pattern (Fig. 6), untreated hippocampal neurons overexpressing mKl displayed an increase in dendritic intersections proximal to the soma (< 50 µm) and a decrease in the number of more distal intersections (Fig. 6G, H). This profile was mildly, but significantly, affected by BDNF treatment with increased number of intersections at approximately 50 µm from the soma (Fig. 6L). Despite this effect, BDNF-treated neurons overexpressing mKl demonstrated a reduced number of intersections when compared to control neurons and neurons overexpressing sKl that were treated with BDNF (Fig. 6I). Untreated neurons overexpressing sKl demonstrated an intermediate phenotype with smaller differences versus control (empty plasmid) neurons (Fig. 6G, H). Treatment of neurons overexpressing sKl with BDNF rescued the dendrite branching pattern to that of control neurons (Fig. 6I, K). In the control condition, BDNF treatment did not have a significant effect when the dendrites were not separated into different orders (Fig. 6G, J).

**Fig. 7:**
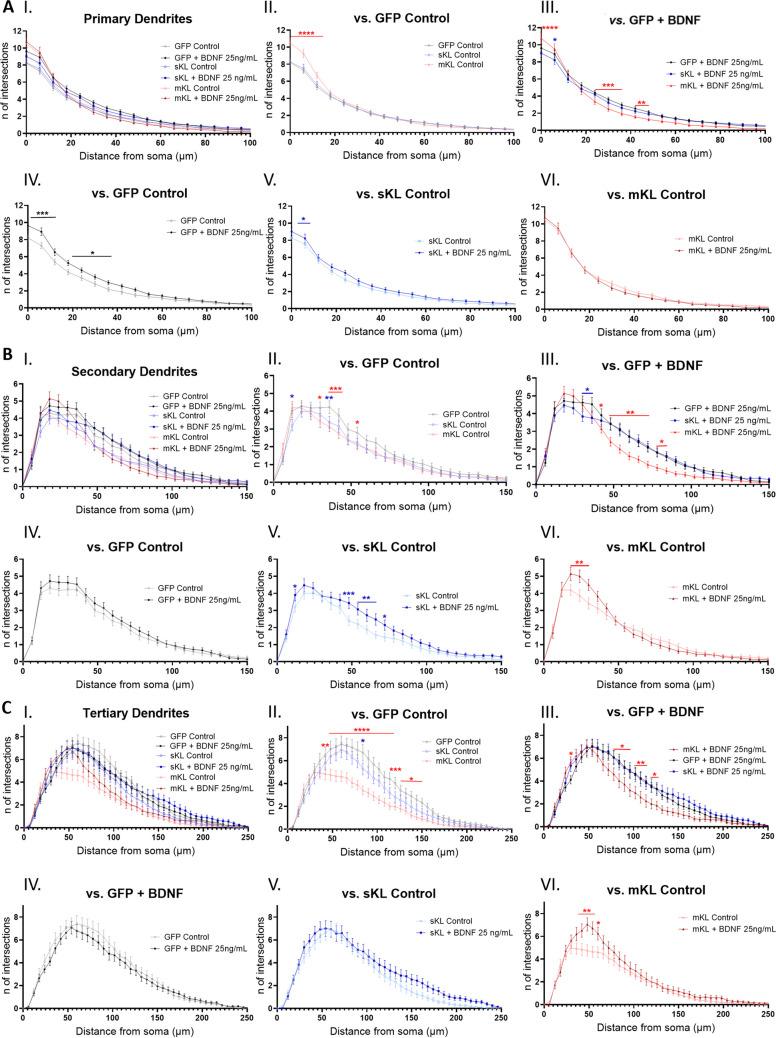
Effects of α-Kl isoform overexpression and BDNF treatment on dendrite order-specific Sholl profiles. Sholl analysis of neurons at DIV 14 that were transfected with empty, sKl, or mKl constructs and untreated (control condition) or treated with 25 ng/mL BDNF for 72 h. **A** Sholl curves of primary dendrites. **B** Sholl curves of secondary dendrites. **C** Sholl curves of tertiary and higher order dendrites. Graph labels: I. All groups; II. Untreated groups; III. BDNF treatment; IV. BDNF treatment of the control (empty) group; V. BDNF treatment on cells overexpressing sKl; VI. Cells overexpressing mKl with no treatment or treatment with BDNF. Data are presented as mean ± S.E.M; *n* = 41–59. *p < 0.05, **p < 0.01, ***p < 0.001, ****p < 0.0001 as determined by two-way ANOVA followed by Tukey’s multiple comparisons test in which black asterisks indicate comparison versus not treated control (empty) group; blue asterisks versus sKl group; red asterisks, versus mKl group

When Sholl analysis was performed for different dendrite orders, BDNF treatment significantly increased primary dendrite intersections in the control group (empty) (Fig. 7A-I, IV), and in neurons overexpressing sKl (Fig. 7A-I, V) with no significant effects on neurons overexpressing mKl (Fig. 7A-I, VI). mKl overexpression significantly increased the number of proximal intersections, while sKl overexpression had no effect (Fig. 7A-II). When we analyzed the impact of BDNF treatment in each condition, the number of primary dendrite intersections was similar in neurons overexpressing mKl and in untreated sister cultures, and overexpression of sKl led to a smaller increase in dendrites promoted by BDNF compared to the control group (Fig. 7A-III-VI).

For secondary dendrites, overexpression of either isoform had a negative impact on dendrite branching (Fig. 7B-I, II). When comparing the effect of α-Kl isoform overexpression on BDNF treatment, neurons overexpressing sKl demonstrated Sholl curves similar to those of control, while neurons overexpressing mKl retained a morphology similar to the untreated mKl overxpression group, with only an increase in proximal branches (Fig. 7B-III-VI).

Overexpression of the mKl isoform significantly decreased the number of tertiary and higher order intersections in untreated cells (Fig. 7C-I, II). Neurons overexpressing sKl presented an intermediate Sholl curve with a minor difference when compared to the empty untreated groups (Fig. 7C-I, II). After BDNF treatment, neurons overexpressing mKl demonstrated an increase in proximal branches compared to untreated neurons overexpressing mKl (Fig. 7C-VI). Interestingly, neurons overexpressing mKl that were treated with BDNF continued to demonstrate a reduced number of intersections compared to the control condition, while neurons with sKl overexpression presented a dendrite branching phenotype similar to that of control neurons (Fig. 7C-III).

Taken together, our results support the idea that mKl and sKl shape the dendritic arbor via distinct mechanisms.

## Discussion

**Fig. 8:**
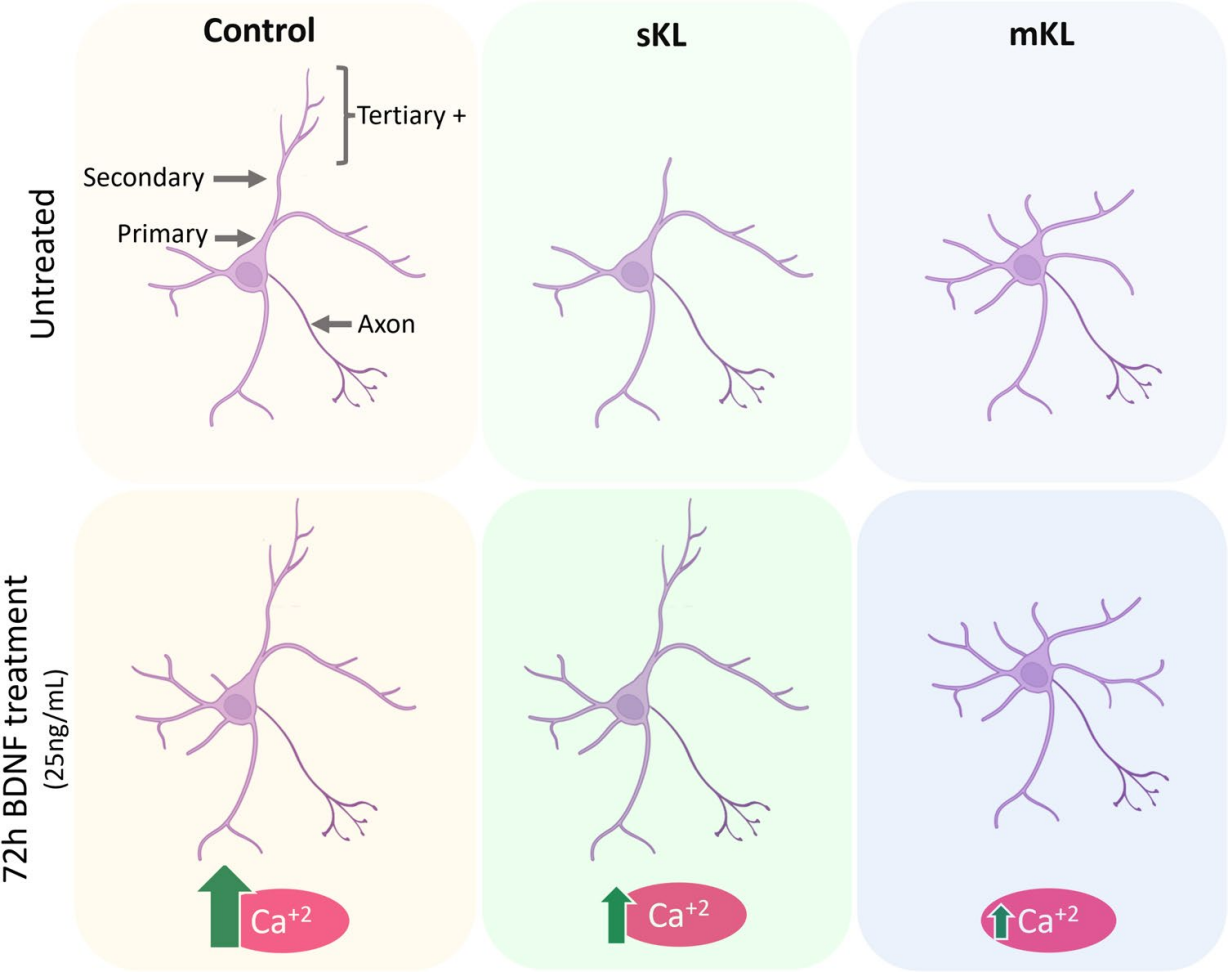
Summary of the effects of α-Kl overexpression on the response to BDNF treatment in primary neurons. sKl and mKl overexpression reduces secondary and tertiary and higher order branching, and mKl increases the number of primary branches in the untreated condition. BDNF treatment for 72 h leads to an increase in primary and secondary dendrites in the control condition and a restoration of the control phenotype in neurons overexpressing sKl. In mKl overexpressing neurons, BDNF promotes a small, but significant, increase in primary and secondary dendrites, but the dendritic arbor is different than that of control neurons. BDNF treatment for 72 h induces ERK phosphorylation and a rise in intracellular calcium levels that are lowered by α-Kl overexpression. Created with BioRender.com

In this study, we present for the first time, evidence that overexpression of α-Kl interferes with growth factor signaling, and specifically BDNF signaling, in neurons and that the mKl and sKl isoforms have distinct impacts on BDNF signaling and changes to dendrite arborization (Fig. 8). Although the effects of mKl and sKl overexpression on dendrite branching partially overlap, they are affected differently by BDNF treatment. Neurons overexpressing sKl display changes in BDNF-promoted increases intracellular levels of Ca^2+^ and changes to dendrite branching, with the latter phenotype restored to control levels after BDNF application. Overexpression of mKl results in a greater change in BDNF-promoted increases in intracellular Ca^2+^ levels; however, the dendrite branching profile in these neurons was affected by BDNF only at the more proximal regions of the dendritic arbor. Thus, even after stimulation with BDNF, neurons overexpressing mKl retain a morphology that resembles immature neurons.

Previous studies have implicated α-Kl in growth factor signaling [11], and others suggest that α-Kl affects neuronal maturation [43, 44, 52]. However, these studies did not investigate the specific effects of α-Kl overexpression in growth factor signaling in the CNS or neuronal cells or the specific effects of the different α-Kl isoforms. The different isoforms have distinct cellular targets [11, 53]. The mKl isoform directly interacts with fibroblast growth factor receptor 1 (FGFR1), acting as a co-receptor for FGF-23. This interaction modulates the activity of FGFR1 and depends on the presence of both KL1 and KL2 domains [53]. The isoforms containing only KL1, either the sKl produced by alternative splicing or the product of mKl cleavage, possess a pocket that can bind to sialic acid present on membrane gangliosides or glycosylated proteins [11, 12]. Another line of evidence suggests that sKl alters protein stability in the membrane by removal of sialic acid residues [9].

Distinct mechanisms may be used by mKL and sKL to regulate dendrite branching. While sKL and cleaved mKL containing only KL1 modulate growth-factor related signaling via inhibition of the PI3K-AKT pathway [3], mKLacts via an additional mechanism that includes direct interaction with FGFR, modifying its specificity towards FGF-23[4].

In this work, we investigated tyrosine kinase receptor signaling, specifically TrKB, ERK, AKT, and CREB phosphorylation and changes to intracellular Ca^2+^ levels. We did not find evidence that stable overexpression of mKL and sKL alters TrKB, ERK, AKT, or CREB phosphorylation after acute (5 min) or prolonged (72 h) BDNF treatment. However, shorter exposure times to mKL and sKL could alter AKT and ERK signaling pathways, as demonstrated by previous publications [1, 2].

The novelty of our data is the effect of treatment with the α-Klotho different isoforms on intracellular Ca^2+^ levels during prolonged BDNF treatment. mKL overexpression has a greater negative impact on BDNF-mediated increases in Ca^2+^ levels after 72 h of treatment (Fig. 3D). After prolonged mKL overexpression, intracellular Ca^2+^ levels do not differ significantly from the levels of untreated control (empty) neurons. In contrast, neurons overexpressing sKL respond to BDNF treatment by increasing the intracellular levels of Ca^2+^ in comparison to basal levels of the untreated control (empty) group. We propose that the ability of neurons that overexpress sKL to maintain higher intracellular Ca^2+^ levels allows for the recovery of the dendrite branching phenotype observed after the 72 h BDNF treatment.

Our results demonstrate that α-Kl isoforms have different expression patterns in the brain. This is not unexpected as different brain regions have distinct anatomical organization and gene expression profiles [54]. It is plausible that the difference in expression could be attributed to the alternative splicing that generates mKl and sKl or to regulation that takes place at the post-translational level. An additional difference between the isoforms is subcellular distribution and processing for release into the extracellular space. As a membrane protein, the release of specific domains of mKl is dependent on tissue expression and activity of secretases. In fact, mKl can be shed by α, β, and y-secretases, and its cleavage can be influenced by hormones, such as insulin [5, 6, 28]. The stimulation of α-secretases ADAM 10 and 17 increases the release of the KL1 and KL2 fragments [6]. For the secreted isoform, the presence of an extra sequence directs this protein into the secretory pathway, and little is known about the factors that influence its release [55].

We observed high α-Kl levels in the hippocampus at postnatal time points that coincide with the critical period for development of this structure, in which neurons present different plasticity mechanisms from that of mature neurons [56, 57]. Thus, the reduction of α-Kl levels after P21 in the hippocampus may be involved in the alterations in plasticity needed for the completion of postnatal maturation.

Importantly, a limitation of our study is that we evaluated the effects of overexpression of the different α-Kl isoforms on dendrite branching. It is possible that this overexpression could result in dominant negative effects. Whether variations in α-Kl levels play a physiological role in dendrite morphogenesis should be addressed by future research. In addition, future studies will determine if the alterations in dendrite branching promoted by α-Kl may offer an advantage in pathological conditions that affect dendrite morphology, such as traumatic brain injury and stroke. The information gained from these studies could lead to new avenues for regaining function of the injured brain.

## Supplementary Information

Below is the link to the electronic supplementary material.

Supplementary file1 (PDF 556 KB)

## Data Availability

Further information and data will be shared via figureshare.com or Box with a link provided upon reasonable request.
