# Supplementary material for: Overexpression of α-Klotho isoforms promotes distinct Effects on BDNF-Induced Alterations in Dendritic Morphology
Source: Mol Neurobiol. 2024 Apr 9;61(11):9155–70. doi: 10.1007/s12035-024-04171-y (PMC11496329; doi:10.1007/s12035-024-04171-y)
Supplement: Supplementary file 1 — Supplementary file1 (PDF 556 KB) [file 12035_2024_4171_MOESM1_ESM.pdf]

Overexpression of α-Klotho isoforms promotes distinct effects on BDNF-induced alterations in dendritic morphology

Marina Minto Cararo-Lopes[1,2*], Ratchell Sadovnik[1] Allen Fu[1], Shradha Suresh[1,3], Srinivasa Gandu[1,2], and Bonnie L. Firestein[1*].

[1]Department of Cell Biology and Neuroscience, Rutgers, The State University of New Jersey, Piscataway, NJ, USA; [2]Cell and Developmental Biology Graduate Program, Rutgers, The State University of New Jersey, Piscataway, NJ, USA; [3]Neuroscience Graduate Program, Rutgers, The State University of New Jersey, Piscataway, NJ, USA;
* Corresponding author: firestein@biology.rutgers.edu

Supplementary Material

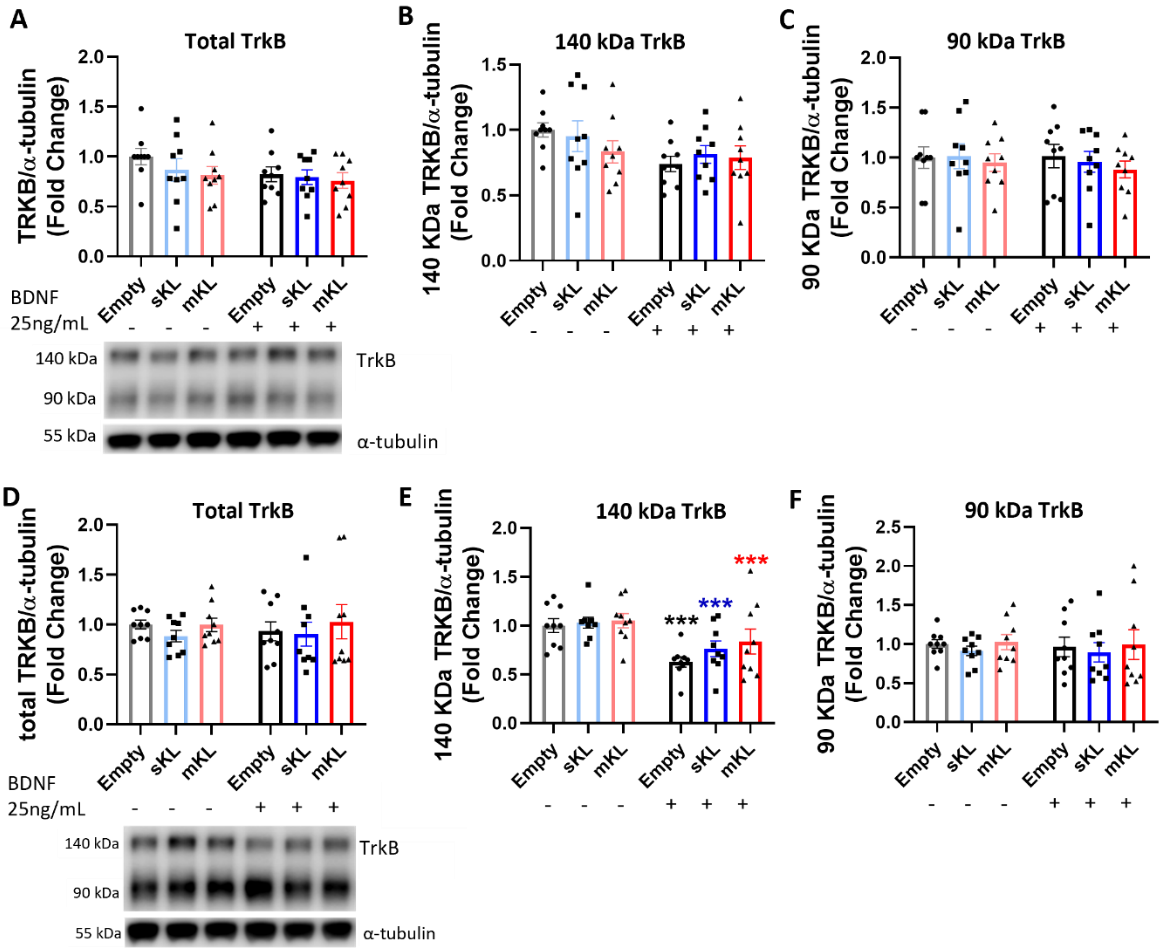

Fig. S1 72 h BDNF treatment reduces the levels of full-length TrkB receptors. Western blot analysis of TrkB levels after (**A-C**) 5 minute and (**D-F**) 72h BDNF treatment. (**A**,**D**) Quantification of 140 kDa full-length and 90 kDa forms of TrkB receptor and respective Western blot images. (**B**,**E**) Quantification of full-length TrkB receptor levels. (**C**,**F**) Quantification 90 kDa form of TrkB receptor levels. Data are presented as mean ± S.E.M. ***p<0.001 as determined by two-way ANOVA followed by Tukey's multiple comparisons test in which black asterisks indicate comparison versus untreated empty group; blue asterisks, versus untreated sKl group; red asterisks, versus untreated mKl group. Data were obtained from a minimum of three independent trials.

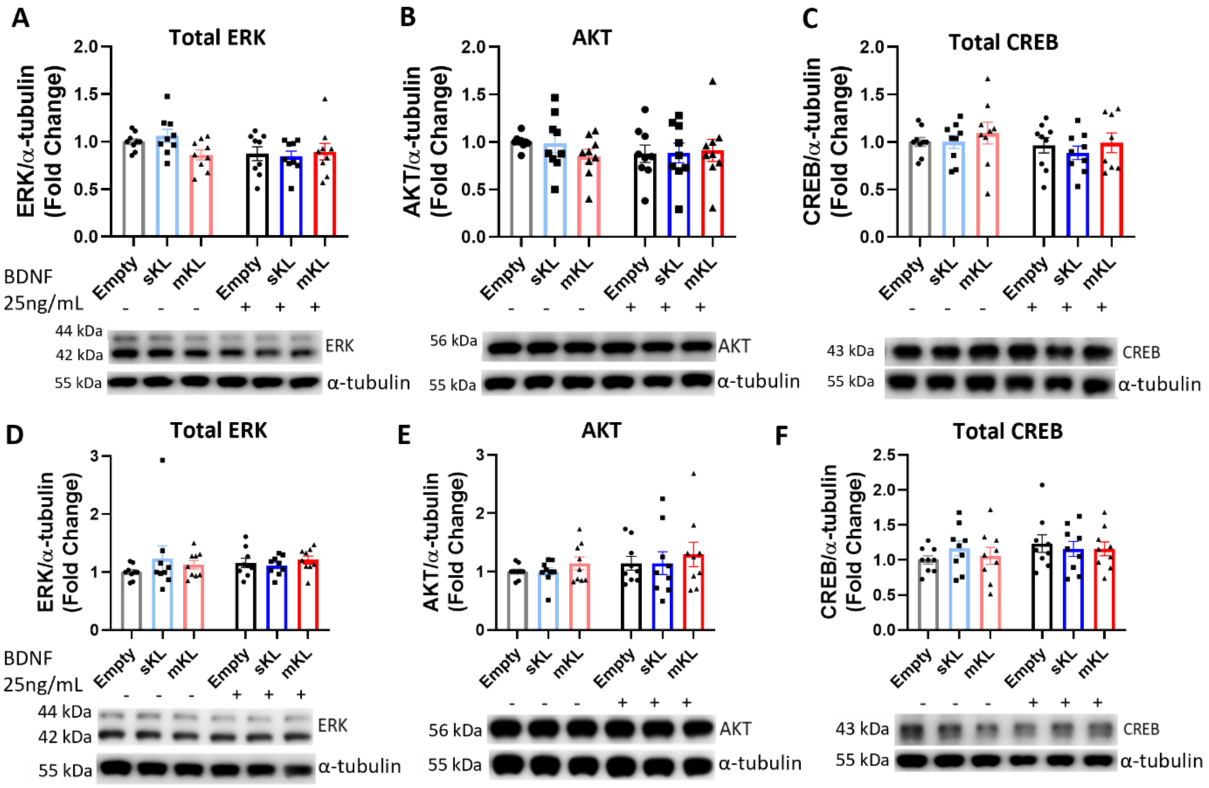

Fig. S2 Neither overexpression of α-Kl isoforms nor BDNF treatment impacts total ERK, AKT, and CREB levels. Analysis of downstream signaling after (**A-C**) 5 minute or (**D-F**) 72h BDNF treatment of cortical cells on DIV 14 previously transduced at DIV 7 with AAV-empty, AAV-sKl, or AAV-mKl vectors. Densitometric quantification and respective representative Western blot images of (**A,D**) ERK, (**B,E**) AKT, (**C,F**) CREB. Levels of protein were normalized to α-tubulin and expressed as fold change of control (empty, untreated) group. Data were obtained from three independent trials and are presented as mean ± S.E.M; No significant differences were determined by two-way ANOVA followed by Tukey's multiple comparisons test.
